# Proteomic identification of galectin-11 and 14 ligands from *Haemonchus contortus*

**DOI:** 10.7717/peerj.4510

**Published:** 2018-03-19

**Authors:** Dhanasekaran Sakthivel, Jaclyn Swan, Sarah Preston, MD Shakif-Azam, Pierre Faou, Yaqing Jiao, Rachael Downs, Harinda Rajapaksha, Robin Gasser, David Piedrafita, Travis Beddoe

**Affiliations:** 1 Department of Animal, Plant and Soil Science and Centre for AgriBioscience (AgriBio), La Trobe University, Bundoora, Victoria, Australia; 2 Department of Biochemistry and Molecular Biology, Monash University, Clayton, Australia; 3 School of Applied and Biomedical Sciences, Federation University, Churchill, Australia; 4 Melbourne Veterinary School, Faculty of Veterinary and Agricultural Sciences, University of Melbourne, Melbourne, Australia; 5 Faculty of Science and Technology, Federation University, Ballarat, Australia; 6 Department of Biochemistry & Genetics, La Trobe Institute for Molecular Science, La Trobe University, Bundoora, Australia

**Keywords:** Mass spectrometry, Host-parasite interactome, Galectin-14, *Haemonchus contortus*, Galectin-11, Gastrointestinal parasite, Galectin

## Abstract

*Haemonchus contortus* is the most pathogenic nematode of small ruminants. Infection in sheep and goats results in anaemia that decreases animal productivity and can ultimately cause death. The involvement of ruminant-specific galectin-11 (LGALS-11) and galectin-14 (LGALS-14) has been postulated to play important roles in protective immune responses against parasitic infection; however, their ligands are unknown. In the current study, LGALS-11 and LGALS-14 ligands in *H. contortus* were identified from larval (L4) and adult parasitic stages extracts using immobilised LGALS-11 and LGALS-14 affinity column chromatography and mass spectrometry. Both LGALS-11 and LGALS-14 bound more putative protein targets in the adult stage of *H. contortus* (43 proteins) when compared to the larval stage (two proteins). Of the 43 proteins identified in the adult stage, 34 and 35 proteins were bound by LGALS-11 and LGALS-14, respectively, with 26 proteins binding to both galectins. Interestingly, hematophagous stage-specific sperm-coating protein and zinc metalloprotease (M13), which are known vaccine candidates, were identified as putative ligands of both LGALS-11 and LGALS-14. The identification of glycoproteins of *H. contortus* by LGALS-11 and LGALS-14 provide new insights into host-parasite interactions and the potential for developing new interventions.

## Introduction

*Haemonchus contortus* is a dominant blood feeding gastrointestinal nematode (GIN) parasite of small ruminants. Blood feeding by *H. contortus* results in haemorrhagic gastritis, oedema, diarrhoea and death in severe infections, leading to significant economic impact through decreased livestock production (Mavrot, Hertzberg & Torgerson, 2015; McLeod, 1995; Roeber, Jex & Gasser, 2013). Sheep can develop effective immunity to *H. contortus* infection and vaccine-induced protection using *H. contortus*-derived molecules such as the H-gal-GP, a gut-derived, galactose-containing glycoprotein complex and integral membrane glycoprotein complex, termed H11, a family of microsomal aminopeptidases have been demonstrated, suggesting that the control of this parasite through vaccination is possible (Nisbet et al., 2016). However, what host molecules recognise these glycoproteins are poorly understood. Recently it has been shown that galectins have been showed to play major roles in host defence against microbial pathogens. Galectins are a family of carbohydrate-binding molecules with characteristic domain organization and affinity for *β*-galactosides that mediates a variety of important cellular functions, including inflammation and immune responses due to binding both self and non-self glycans (Vasta et al., 2017).

In particular, ruminants highly upregulate the expression of two specific galectins (LGALS-11 and LGALS-14) upon infection by various parasites such as *Ostertagia ostertagi, Cooperia oncophora* and *H. contortus* (Dunphy et al., 2000; Dunphy et al., 2002; Hoorens et al., 2011; Meeusen, Balic & Bowles, 2005). LGALS-14 is secreted by eosinophil immune cells that are critical for immunity through killing the larval stages of *H. contortus* (Balic, Cunningham & Meeusen, 2006; Dunphy et al., 2002; Young et al., 2009). LGALS-14 is thought to be the homologue of human galectin-10, which is also secreted by eosinophils (Ackerman et al., 2002). Analysis of *H. contortus* infected sheep demonstrated release of LGALS-14 into the gastrointestinal mucus, the interface of host and parasite interaction (Dunphy et al., 2002). In addition, kinetic studies of LGAL-14 showed that release into the mucus occurred soon after challenge infection, and correlated with a reduction in parasitic burden (Robinson et al., 2011). Additionally, it has been shown that LGAL-14 can bind directly to another parasite *Fasciola hepatica* suggesting it can inhibit infection (Young et al., 2012).

The second galectin (LGALS-11) is specifically expressed and secreted during *H. contortus* infections in previously infected sheep that had developed resistance to the parasite (Dunphy et al., 2000). Immunohistochemistry revealed that LGALS-11 was secreted by epithelial cells lining the gastrointestinal tract, where it was localised to the nucleus and cytoplasm of cells. Analysis of the mucosal contents lining the gastrointestinal tract also revealed secretion of LGALS-11 into the mucus. An observation of increased mucus stickiness corresponding with the production of LGALS-11 suggested that it might work by interacting with the mucus to impede *H. contortus* motility (Robinson et al., 2011). Recent immunofluorescent staining techniques using a recombinant form of galectin-11 have revealed binding to the fourth larval stage and adult *H. contortus* that has resulted in impaired development. These studies suggest a more direct or additional role for LGALS-11 during *H. contortus* infections.

It appears that both LGALS-11 and LGALS-14 mediate critical immune regulatory effects and/or mediate direct parasite stage-specific killing (Haslam et al., 1998; Preston et al., 2015b). Although the interactions of these host galectin-parasite glycoconjugates are likely to be critical for parasite control, the parasite glycoconjugate molecules that they recognise are unknown. For the first time, this study describes the ligands of sheep LGALS-11 and LGALS-14 in larval and adult stages of *H. contortus*.

## Materials and Methods

### Preparation of L4 larvae and collection of adult parasites

*H. contortus* (Haecon-5 strain) was maintained in Professor Gasser’s laboratory, Melbourne Veterinary School, The University of Melbourne and was used in this study. Mature fourth stage larvae (L4 stage) and adults of *H. contortus* were prepared using established protocols (Preston et al., 2015a). Briefly, third-stage larvae (L3) were isolated from faeces from *H. contortus*-infected sheep. The cuticle was removed from the L3s by using sodium hypochlorite, the exsheathed L3 (xL3) worms were washed three times with 0.9% (w/v) normal saline. Approximately 2,000 xL3/ml worms were resuspended in Dulbecco’s modified Eagle Medium+GlutaMax (DMEM) (Gibco-Invitrogen, Gaithersburg, MD, USA) containing 10,000 IU/ml of penicillin and 10,000 µg/ml of streptomycin (Gibco-Invitrogen, Gaithersburg, MD, USA) and 0.5% (v/v) amphotericin (GE Healthcare, Chicago, IL, USA). Medium containing xL3s was incubated at 37 °C with 10% (v/v) CO_2_ for 7 days. Fresh DMEM was substituted at two-day intervals and larval development was examined each day. The xL3 and L4 stages were differentiated based on distinctive morphological characteristics (see Preston et al., 2015a). Animal experimental procedures were approved by the Monash University Animal Ethics Committee (Ethics # SOBSA/P/2009/44). Adults of *H. contortus* were collected from Merino ewes (8–12 months old) which were experimentally infected with 10,000 L3s and the infected animals were euthanised 52 days post infection by injection of pentobarbitone (Lethobarb^®^, Virbac Pty Ltd, Australia). Approximately 5,000 adult worms of mixed sex were collected from the abomasal content and washed five times with 0.9% (v/v) biological saline (Baxter, Australia). Immediately after washing, the worms were snap frozen in liquid nitrogen and stored at −80 °C until further use.

### Total larval protein lysate preparation

Lysates were prepared using radioimmunoprecipitation assay buffer (RIPA) as previously described with minor modifications (Maduzia, Yu & Zhang, 2011). Briefly, 500 mg of larval or adult *H. contortus* were incubated with 100 mM β-D-galactose containing 0.9% (v/v) normal saline for 12 h to remove native galectins (bound to the adult parasite surface recovered from infected sheep) and washed three times with normal saline. Larval or adult *H. contortus* were then resuspended in 5 ml of ice-cold RIPA buffer (20 mM Tris-HCL pH 7.2, 100 mM NaCl, 1% (v/v) Nonidet P-40, 0.1% (w/v) sodium deoxycholate (DOC), 0.05% (w/v) sodium dodecyl sulphate (SDS), 1% (v/v) Triton X-100, 10 mM TCEP (Tris (2-carboxyethyl) phosphine)) and lysed by sonication (30 s, 8 times with three min interval at 40% amplitude). Cellular debris was removed by centrifugation (15,000 × *g* for 20 min) at 4 °C, and any particles in the supernatant removed by filtering through a 0.22 µm filter. Lysates were dialysed using 3 kDa molecular weight cut-off against binding buffer ((20 mM Tris–HCl pH 7.5, 100 mM NaCl, 0.5% (v/v) Nonidet P-40, 0.1% (w/v) DOC, 0.05% (w/v) SDS, 1% (v/v) Triton X-100, 10 mM TCEP)).

### LGALS-11 and LGALS-14 affinity column

**Figure 1:**
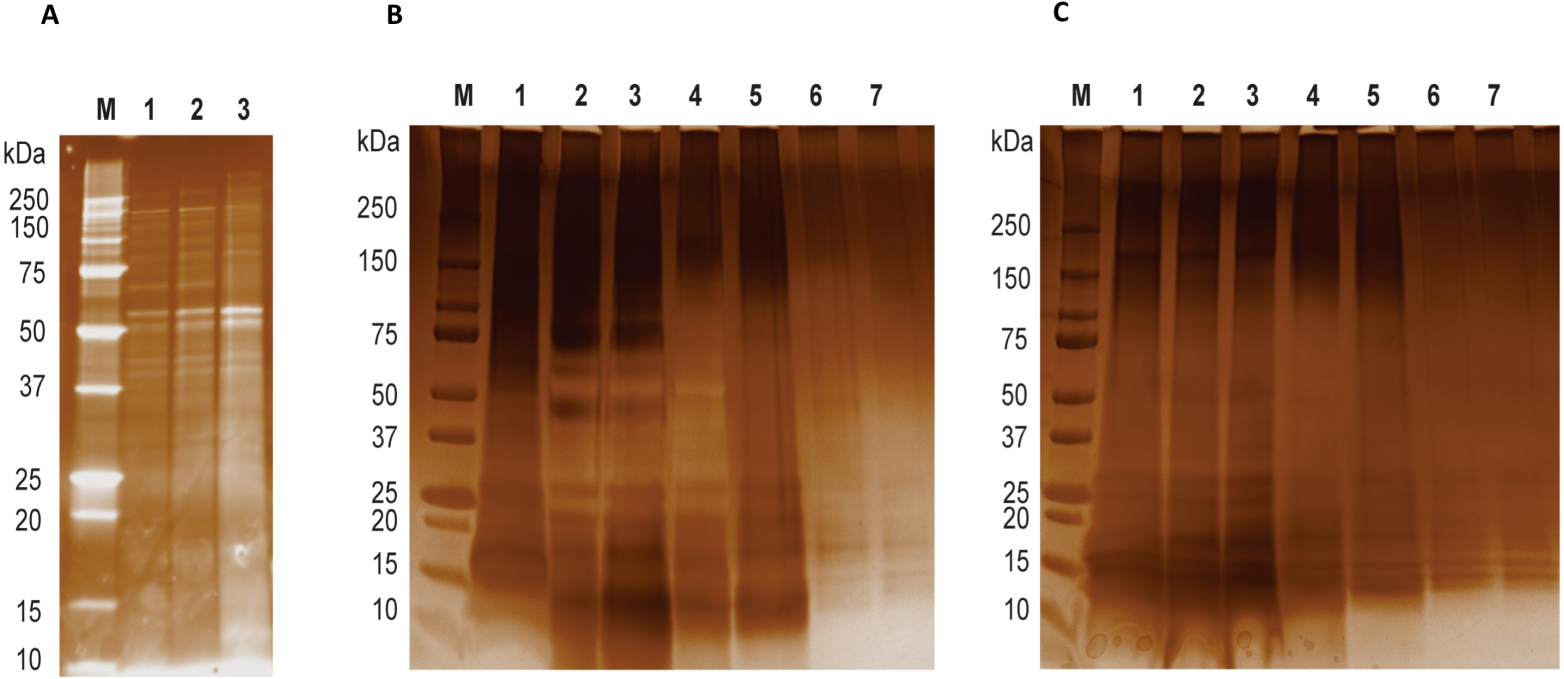
LGALS-11 and LGALS-14 interactome. (A) Protein profiling of larval and adult stages of *Haemonchus contortus*. (M) Molecular weight markers; (Lane 1 & 2) lysates prepared from L4 stage; (Lane 3) lysates prepared from adult stage. (B) Protein profile of adult stage parasite bound to LGALS-11 and LGALS-14 and (C) larval stage parasite bound to LGALS-11 and LGALS-14. M) Molecular weight markers; (Lane 1) total parasite lysate, (Lane 2 & 3) unbound protein fractions of LGALS-11 and LGALS-14 column, (Lane 4 & 5) column wash of LGALS-11 and LGALS-14 column and (Lane 6 & 7) eluted protein of LGALS-11 and LGALS-14 column.

Recombinant LGALS-11 and LGALS-14 were expressed and purified as described previously (Sakthivel et al., 2015; Fig. 1). The recombinant protein (5 mg/ml) was buffer-exchanged into HEPES buffer (10 mM HEPES-NaOH pH 7.5, 100 mM NaCl, 10 mM TCEP) and immobilised by coupling to N-hydroxysuccinamide (NHS)-activated Sepharose (GE Healthcare, Chicago, IL, USA) following the manufacturer’s protocol. Briefly, 4 ml of NHS-activated Sepharose was washed with 15 column-volumes of ice-cold 1 mM HCl. The washed Sepharose beads were equilibrated with 20 ml of coupling buffer (10 mM HEPES-NaOH pH 7.5, 100 mM NaCl, 10 mM TCEP). Following equilibration, LGALS-11 and LGALS-14 were added separately to the activated Sepharose and allowed to couple for 5 h at 22 °C. Following the coupling reaction, the unused, activated sites were blocked using 15 column-volumes of blocking buffer (100 mM Tris–HCl pH 8.0, 100 mM NaCl) for 3 h. Following blocking, the Sepharose beads were washed alternatively six times with 15 column-volumes of 100 mM Tris-HCL pH 8.0 followed by 100 mM sodium acetate pH 5.0 and then 250 mM NaCl. The galectin affinity column was maintained in storage buffer (20 mM Tris–HCl pH 8.0, 100 mM NaCl, 10 mM TCEP, NaAc 0.02% (w/v)) until further use. A control resin was also prepared without any protein ligand.

### Isolation of LGALS-11 and LGALS-14 parasite ligands

Immobilised LGALS-11, -14 or control slurry (1 ml) was loaded into individual columns. Larval and adult *H. contortus* lysates (150 mg) were diluted with 5 ml of binding buffer (20 mM Tris–HCl pH 7.5, 100 mM NaCl, 0.5% (v/v) Nonidet P-40, 0.1% (w/v) DOC, 0.05% (w/v) SDS, 1% (v/v) Triton X-100, 10 mM TCEP) and applied to the galectin affinity column and incubated for 16 h at 4 °C. Thereafter, columns were washed three times with 15 ml of RIPA buffer, the captured protein fractions were eluted by incubating for 2 h with galactose elution buffer (250 mM β-D-galactose 20 mM Tris–HCl pH 8.0, 100 mM NaCl, 10 mM TCEP) and the resultant supernatant was subjected to LC-MS/MS analysis to identify the protein molecules present. The eluted protein products were analysed by 12% SDS-PAGE stained with nitrate. The unbound fractions, column wash and eluted proteins fractions were concentrated using sodium deoxycholate/trichloroacetic acid precipitation method to allow the visualisation of protein products as previously described (Arnold & Ulbrich-Hofmann, 1999).

### Mass spectrometry (ESI–LC–MS/MS) analysis of galectin binding proteins

Eluted protein samples were dissolved in digestion buffer (8 M urea, 50 mM NH_4_HCO_3_, 10 mM dithiothreitol) and incubated at 25 °C for 5 h. Following incubation, iodoacetamide (IAA) was added to final concentration of 55 mM to alkylate thiol groups and incubated for 35 min at 20 °C in the dark. The alkylated protein preparation was diluted with 1M urea in 25 mM ammonium bicarbonate (pH 8.5) and sequencing-grade trypsin (Promega) was added to a final concentration of 5 µM. The reaction was incubated for 16 h at 37 °C in the dark. The digests were acidified with 1% (v/v) trifluoroacetic acid (TFA) and the peptides desalted on poly(styrene-divinylbebzebe) copolymer (SDB) (Empore) StageTips as described previously (Rappsilber, Mann & Ishihama, 2007).

Trypsin-digested peptides were reconstituted in 0.1% (v/v) TFA and 2% (v/v) acetonitrile (ACN) and then loaded onto a guard column (C_18_ PepMap 100 µm ID × 2 cm trapping column; Thermo-Fisher Scientific, Waltham, MA, USA) at 5 µl/min and washed for 6 min before switching the guard column, in line with the analytical column (Vydac MS C_18_, 3 µm, 300 Å and 75 µm ID × 25 cm). The separation of peptides was performed at 300 nl/min using a non-linear ACN gradient of buffer A (0.1% (v/v) formic acid, 2% (v/v) ACN) and buffer B (0.1% (v/v) formic acid, 80% (v/v) ACN), starting at 5% (v/v) buffer B to 55% for 120 min. Data were collected on an Orbitrap Elite (Thermo-Fisher Scientific, Waltham, MA, USA) in a data-dependent acquisition mode using m/z 300–1,500 as MS scan range, CID MS/MS spectra and were collected for the 20 most intense ions. Dynamic exclusion parameters were set as described previously (Nguyen et al., 2016). The Orbitrap Elite was operated in dual analyser mode, with the Orbitrap analyser being used for MS and the linear trap being used for MS/MS. Pull-down and LC-MS/MS analysis were performed three times on different days.

### Database search and protein identification

The MS/MS spectra obtained from the Orbitrap analyser was used to interrogate the Swiss-Prot *Haemonchus contortus* FASTA database (downloaded on 07.31.2016, 21,201 protein entries) in conjugation with common contaminants by use of the Mascot search engine (Matrix Science Ltd., London, UK) as described previously (Perkins et al., 1999). Briefly, carbamidomethylation of cysteines was set as a fixed modification, acetylation of protein N-termini, methionine oxidation were included as variable modifications. Precursor mass tolerance was 10 ppm, product ions were searched at 0.5 Da tolerances, minimum peptide length defined at 6, maximum peptide length 144, and peptide spectral matches (PSM) were validated using Percolator based on q-values at a 1% false discovery rate (FDR). Both peptide and protein identifications were reported at a false discovery rate (FDR) of 1%. The mass spectrometry proteomics data have been deposited to the ProteomeXchange Consortium via the PRIDE partner repository with the data set identifier PXD008435 and DOI 10.6019/PXD008435.

### Protein-protein interaction analysis and visualisation

Normalized spectral abundance factor (NSAF) values were calculated for the identified proteins using the Scaffold software v4.7.2 (Searle, 2010). Then proteins were subjected to the significance analysis of interactome (SAINT) (Choi et al., 2011) to identify *bona fide* protein-protein interactions after removing all zero or missing rows. Proteins with a SAINT probability greater than 0.9 were selected as high probability interactions. Finally, the resulting interaction network was visualised using the Cytoscape v3.4.0 (Shannon et al., 2003).

### Analysis of glycosylation

The N- and O-linked glycosylation pattern and the signal peptides of eluted proteins were analysed following the instructions provided in the glycosylation analysis server. Briefly, N-glycosylation and signal peptide was analysed using NetNGlyc 1.0 server (http://www.cbs.dtu.dk/services/NetNGlyc/). Whereas the O-glycosylation pattern was analysed using NetOGlyc 4.0 Server (http://www.cbs.dtu.dk/services/NetOGlyc/). The results obtained from the N and O glycosylation servers were provided as supplementary results.

## Results and Discussion

### Identification of proteins from *H. contortus* that interact with LGALS-11 and LGALS-14

**Figure 2:**
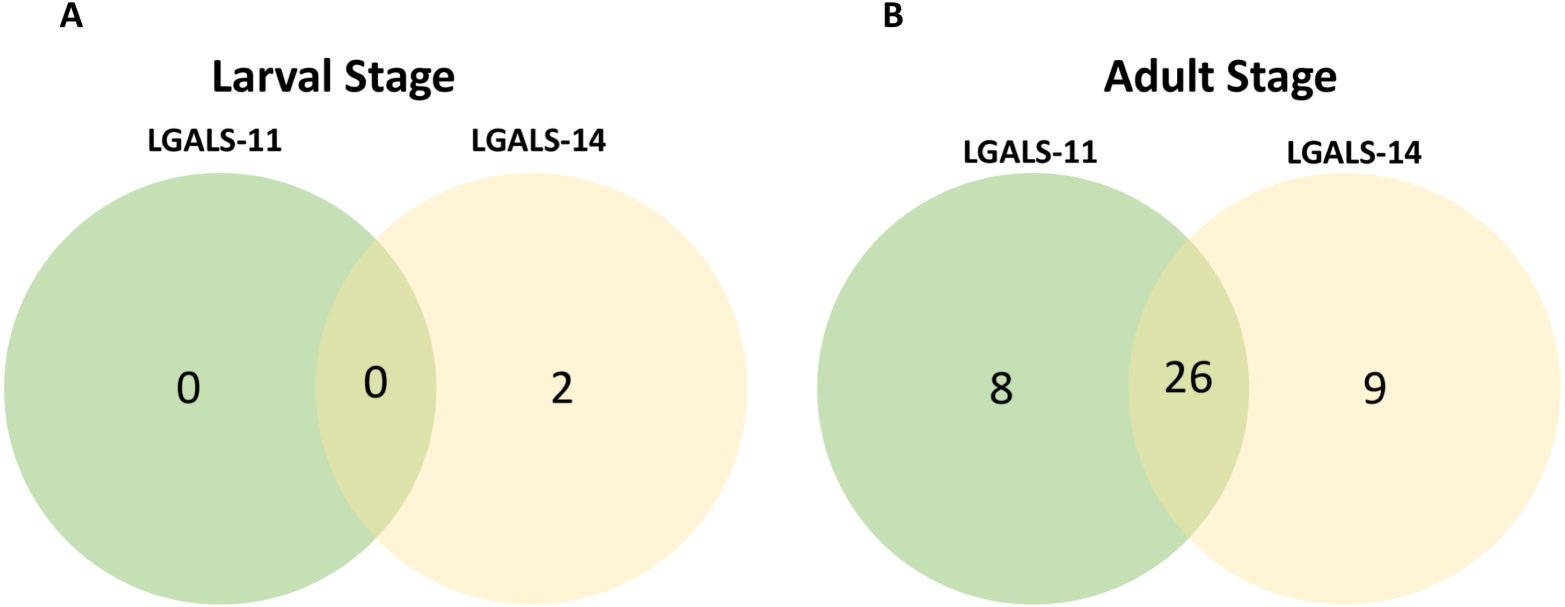
Venn diagram of parasite proteins bound by host galectins. Venn diagram showing the distribution of proteins of the larval (A) and adult (B) stages of *Haemonchus contortus*. In larval and adult stages, 0 and 26 proteins were bound by both the galectins respectively.

Affinity purification and identification of *H. contortus* proteins interacting with LGALS-11 and LGALS-14 is summarized in Fig. S1. Lysates of L4 and adult stages were assessed before loading onto the columns containing the Sepharose immobilised LGALS-11 and LGALS-14 and are shown in Fig. 1A. Multiple bands were observed, with both larval and adult lysates containing a broad range of proteins of differing molecular weights. Following the application of L4 and adult lysates to affinity columns, containing immobilised galectins, bound parasite molecules were eluted with elution buffer-containing galactose (Figs. 1B and 1C). The eluted molecules from both affinity columns and a control column were subjected to LC-MS/MS. Proteins that were identified in two of the three biological replicates were included for further analysis and the proteins that were bound to control resin (Table S1) were removed from the analysis. Overall, 43 individual proteins were identified and grouped based on their respective known or putative biological function(s) (Table 1). The greatest number of proteins identified was in the adult stage of *H. contortus*; with 34 proteins binding to LGALS-11 and 35 proteins binding to LGALS-14. Of those identified proteins, 26 proteins were found to bind to both LGALS-11 and LGALS-14 (Fig. 2). In the L4 larval stage, LGALS-11 and LGALS-14 could bind to 0 and two proteins, respectively.

**Table 1:** Identification by mass spectroscopy of larval and adult *Haemonchus contortus* proteins eluted from LGALS-11 and LGALS-14 columns.

Accession code	Gene description	CV	PSMs	UP	Mascot score	Groups				Log-odds	NSAF	MW (kDa)	Signal peptide	Number of N-and (blue)/ or O-glycosylation sites (red)^a^
						1	2	3	4					
**Metabolic process**														
W6NE18	Peptidase S28 GN=HCOI_01497800	45.21	634	20	7,058	No	Yes	Yes	Yes	145.70	0.071	67.1	No	1/5
W6NFG0	Alpha beta hydrolase fold-1 GN=HCOI_00457700	58.65	504	16	6,681	No	Yes	Yes	Yes	128.68	0.129	36.6	Yes	1/0
W6NFT9	Peptidase S28 GN=HCOI_01562400	23.27	222	18	3,151	No	Yes	No	Yes	11.26	0.0062	128.6	Yes	7/35
W6NJ96	Carboxyl transferase GN=HCOI_00766200	23.39	123	10	1,146	No	Yes	No	Yes	16.01	0.0103	56.1	No	0/7
W6NLA8	Glutamate phenylalanine leucine valine dehydrogenase GN=HCOI_01838800	42.50	102	10	957	No	Yes	No	Yes	20.61	0.020	26.8	No	0/0
W6NG90	Peptidase S28 GN=HCOI_01624000	4.88	101	3	1,218	No	Yes	No	Yes	29.6	0.003	117.0	Yes	4/7
W6NC58	Aldo keto reductase GN=HCOI_00043700	39.64	83	10	789	No	Yes	No	Yes	−0.18	0.009	38.0	No	3/6
W6NAV8	Aldo keto reductase GN=HCOI_00043500	27.22	46	6	477	No	Yes	No	Yes	−0.18	0.0028	40.0	No	2/4
W6NU27	Carboxyl transferase GN=HCOI_00766300	25.83	67	6	909	No	Yes	No	Yes	4.35	0.0023	42.2	No	1/0
W6NKM1	Succinate dehydrogenase iron-sulfur subunit, GN=HCOI_01735500	32.61	64	8	521	No	Yes	No	Yes	34.83	0.0067	31.6	No	1/10
W6NF70	Deoxynucleoside kinase GN=HCOI_01673500	35.42	42	6	242	No	No	No	Yes	−0.18	0.0012	34.8	No	0/0
U6NNG6	Ribosomal protein L7 L12 GN=HCOI_00340500	11.41	30	1	783	No	Yes	No	Yes	−0.18	0.013	19.5	No	1/3
W6NA79	Zinc metallopeptidase M13 GN=HCOI_01030800	37.90	28	3	330	No	No	No	Yes	4.35	0.0067	14.6	No	0/2
W6ND82	von Willebrand factor domain containing protein GN=HCOI_01354500	13.82	26	3	371	No	No	No	Yes	16.01	0.0077	27.5	Yes	2/0
W6NMI7	Proteinase inhibitor I33 GN=HCOI_02015200	18.58	22	5	162	No	No	No	Yes	−0.18	0.0020	25.1	Yes	0/7
W6NKG5	Ribosomal protein L15 GN=HCOI_01717500	5.88	4	1	26	No	No	No	Yes	5.67	0.0002	24.3	No	1/10
W6NEW9	Amidinotransferase GN=HCOI_01556200	23.35	24	4	109	No	Yes	No	No	−0.18	0.0014	29.2	No	0/0
W6NI22	Adenylosuccinate lysase GN=HCOI_00436500	6.41	14	3	141	No	Yes	No	No	12.23	0.0005	76.9	No	1/4
W6NF84	Short-chain dehydrogenase reductase GN=HCOI_01467500	3.07	11	1	137	No	Yes	No	Yes	1.71	0.0003	27.5	No	0/0
W6ND43	Acetyltransferase component of pyruvate dehydrogenase complex GN=HCOI_00576100	1.22	8	1	51	No	Yes	No	No	1.68	0.0001	78.3	No	1/40
**Regulation of biological processes**														
W6NC73	ATPase GN=HCOI_02138200	22.64	23	2	418	No	Yes	No	Yes	−0.18	0.004	17.8	No	1/0
W6NJ12	Filament domain containing protein GN=HCOI_02013700	14.09	20	3	291	No	Yes	No	Yes	−0.18	0.0028	34.2	No	0/4
W6NM02	Fumarate lyase GN=HCOI_01914600	4.73	8	1	62	No	Yes	No	No	4.61	0.0003	29.7	No	1/12
W6NGK5	CRE-DHS-15 protein GN=HCOI_00341400	29.31	6	1	210	No	No	No	Yes	−0.18	0.0031	6.1	No	0/1
W6NI80	Acyl-CoA-binding protein GN=HCOI_01539100	20.69	6	1	205	No	Yes	No	Yes	6.69	0.0018	9.5	No	0/1
W6NCC1	NIPSNAP GN=HCOI_01963300	7.63	6	1	75	No	Yes	No	No	−0.18	0.0013	26.5	No	0/6
W6NWY9	Porin domain containing protein GN=HCOI_01573900	43.97	82	9	1,149	No	Yes	No	Yes	4.35	0.011	30.2	No	3/0
W6NAW4	FG-GAP and Integrin alpha-2/Integrin alpha chain GN=HCOI_01903100	5.90	33	5	340	No	Yes	No	Yes	−0.18	0.0028	133.9	Yes	5/18
W6NAL4	Heat shock protein 70 GN=HCOI_00589700	7.24	9	1	82	No	Yes	No	No	7.23	0.0002	72.4	No	3/7
**Transport**														
W6NVQ1	Lipid transport protein and Vitellinogen and von Willebrand factor domain GN=HCOI_01683400	23.18	209	24	1,465	No	Yes	No	Yes	9.62	0.0039	156.0	No	0/11
W6N9I2	Lipid transport protein GN=HCOI_00072100	35.97	139	13	1,313	No	Yes	No	Yes	51.51	0.0136	56.8	Yes	0/2
W6NQZ5	Mitochondrial substrate solute carrier GN=HCOI_01092000	23.10	53	8	334	No	No	No	Yes	6.2	0.0033	36.3	No	0/3
**Cytoskeleton**														
W6NAH7	Nematode cuticle collagen and Collagen triple helix repeat GN=HCOI_00810700	5.60	85	2	1,399	No	Yes	No	Yes	4.35	0.0117	32.8	No	0/15
W6NHH0	Annexin GN=HCOI_01003500	14.01	22	3	135	No	Yes	No	No	−0.18	0.0008	29.4	No	0/1
W6NE41	Myosin tail GN=HCOI_01216000	3.99	17	3	59	No	Yes	No	No	13.42	0.0008	54.7	No	0/15
W6NF56	Myosin tail GN=HCOI_01461300	34.48	51	8	675	No	Yes	No	Yes	6.2	0.0040	36.5	No	0/14
**Immunomodulatory**														
W6NGA7	SCP extracellular domain GN=HCOI_01577700	14.95	4	2	59	No	Yes	No	Yes	−0.18	0.0011	22.2	No	1/1
**Unknown**														
W6NAN9	CBN-MLC-3 protein GN=HCOI_01274700	49.67	63	7	465	No	Yes	No	Yes	2.32	0.0086	17.0	No	0/2
W6NX42	Uncharacterized protein GN=HCOI_01051700	25.56	38	3	607	No	No	No	Yes	19.09	0.0066	25.5	No	4/40
W6NFI4	Protein C15F1.2 GN=HCOI_01126300	34.87	20	4	97	No	Yes	No	Yes	−0.18	0.0022	27.1	Yes	0/13
W6NB42	Protein C23H5.8, isoform-c GN=HCOI_00648100	14.43	16	2	170	No	Yes	No	Yes	10.80	0.0025	22.5	Yes	1/1
W6NPK3	Uncharacterized protein GN=HCOI_00260500	5.20	10	1	45	No	No	No	Yes	−0.18	0.0001	22.6	Yes	3/11
W6NUX4	Uncharacterized protein GN=HCOI_01608400	4.04	12	1	43	No	Yes	No	Yes	4.04	0.0002	21.8	Yes	0/6

**Notes.**

GN Gene name CV Coverage PSMs Peptide spectrum matches UP Unique peptides Log-odds 0 50% chance Positive values More than 50% Negative values Less than 50%

Groups: 1, LGALS-11 bound protein from L4 larval stage of *H. contortus*; 2, LGALS-11 bound protein from adult stage of *H. contortus*; 3, LGALS-14 bound protein from L4 larval stage of *H. contortus*; 4, LGALS-14 bound protein from adult stage of *H. contortus*.

a Predicated glycosylation sites as determined in materials and methods.

NSAF, Normalized Spectral Abundance Factor.

### Composition of LGALS-11 and LGALS-14 ligands

**Figure 3:**
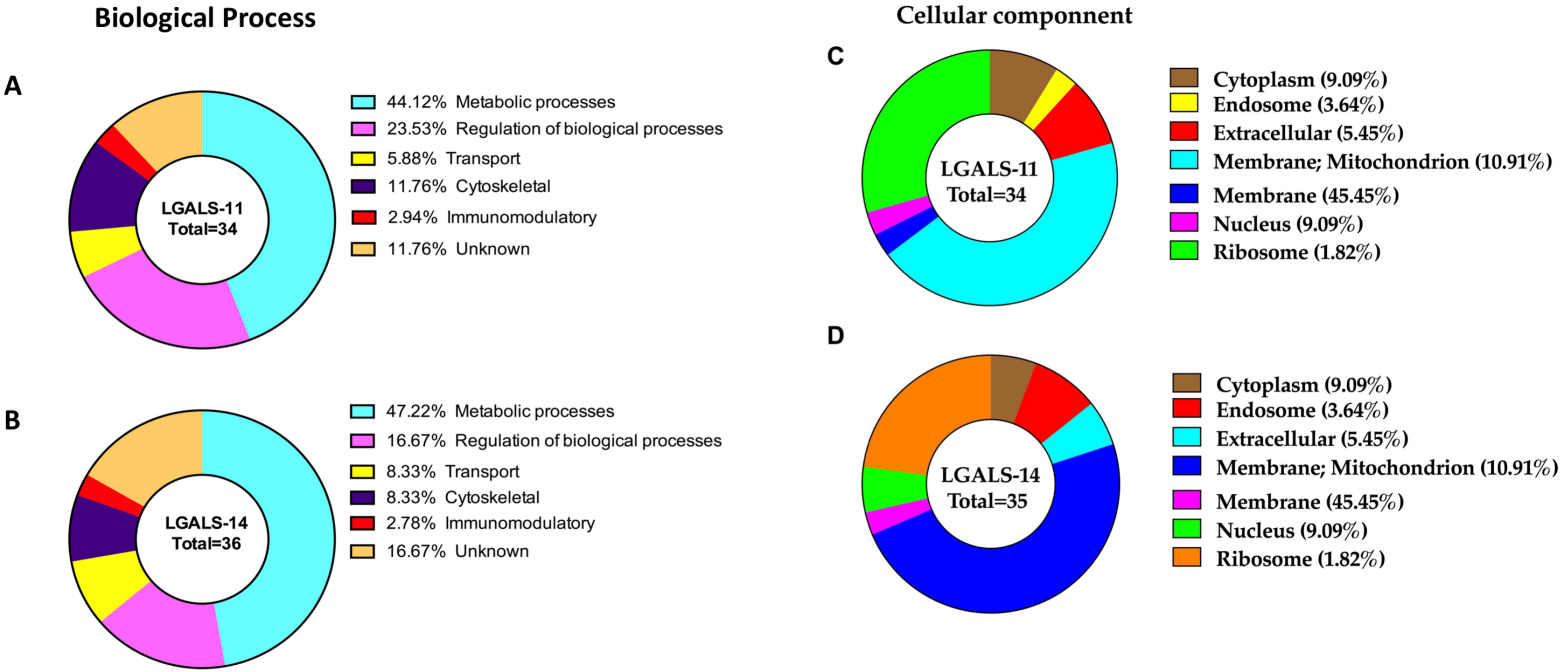
Categorisation of proteins in the adult stage of *Haemonchus contortus* that interacted with host galectins. The profiles were categorised based on biological process of LGALS-11-bound proteins (A) and LGALS-14 bound-proteins (B) and cellular location of LGALS-11-bound proteins (C) and LGALS-14-bound proteins (D).

Approximately 69% of proteins in *H. contortus* that bound specifically to LGALS-11 and/or LGALS-14 were inferred to be involved in metabolic and regulatory processes (Table 1, Fig. 3). Most of these proteins (∼70%) were likely involved in metabolic activities, such as energy metabolism, transcription and translation. These proteins predominantly included regulatory enzymes, such as peptidases, carboxyl transferases, aldo-keto reductases, deoxynucleoside kinase, dehydrogenase, amidinotransferase and RNA polymerase. Another protein group (∼9%) identified represented structural proteins, such as actin, myosin and collagen from *H. contortus* (Table 1, Fig. 3). Other proteins identified had putative roles in molecular transport (e.g., lipid and amino acid transport) or had no assigned function(s) (Fig. 3). *In silico* analysis revealed that approximately 65% of the proteins of the adult stage*,* that bound specifically to LGALS-11 and LGALS-14 had one or more potential glycosylation site (Table 1). On the contrary, about 35% of adult stages specific proteins that bound to LGALS-11 and LGALS-14 were predicted as non-glycosylated. Though animal lectins have a primary preference for glycoconjugates, it is believed that the LGALS-11 and LGALS-14 might also display a glycan independent protein-protein interaction activity similar to previously reported for galectin-1 and galectin-3 (Bawumia et al., 2003; Camby et al., 2006; Menon, Strom & Hughes, 2000; Paz et al., 2001). Galectin-3 has also been shown to interact with proteoglycans which are proteins which have covalently attached glycosaminoglycan chain (Talaga et al., 2016). Most mammalian proteoglycans are major component of extracellular matrix such collagen. A nematode cuticle collagen and collagen triple helix repeat protein (W6NAH7) was identified in adult stages by LGALS-11 and LGALS-14 suggesting that LGALS-11 and LGALS-14 can interact with proteoglycans.

### Larval and adult ligands of LGALS-11 and LGALS-14

More putative ligands (*n* = 43) were identified in the adult stage of *H. contortus* compared with larval stages (*n* = 2) following galectin pull-down assays. Although the L4 stage is a histotropic stage (in glands of the stomach) and would be expected to be in intimate contact with inflammatory mediators, including galectins, it moults (with a change in antigenic profile) within 48–72 h into the immature adult (Meeusen, Balic & Bowles, 2005). This would be expected to limit the antigenic exposure of these parasite antigens to the host. Compared to the adult stage that is relatively long-lived (6–8 weeks), allowing a sustained interaction of host molecules with parasite antigens (Nikolaou & Gasser, 2006; Veglia, 1915). This interaction might be reflected in the specific and localised binding of LGALS-11 in the larvae and the significant staining of LGALS-11 on the surface of adult *H. contortus* (see Preston et al., 2015b). In addition, the L4 stage is relatively small (750–850 µm long), whereas the adult stage is usually 10–30 mm long.

**Figure 4:**
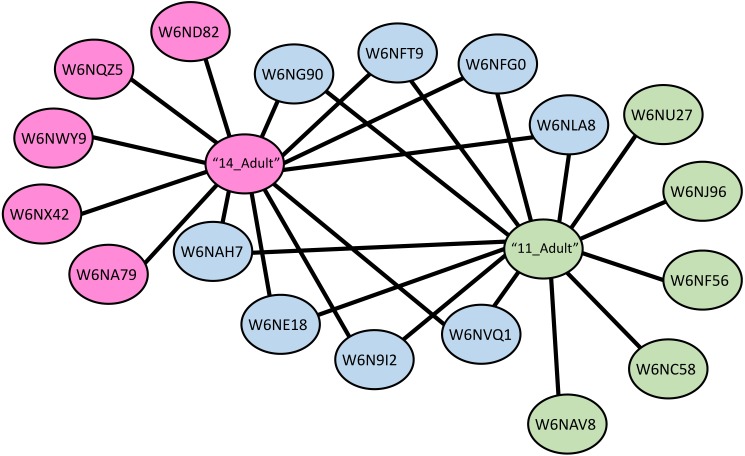
Protein-protein interaction network of adult stage of *Haemonchus contortus* with host galectins. Protein interactions was determined using the software (SAINT) (Choi et al., 2011) and resulting interaction network was visualised using the Cytoscape v3.4.0.

A protein-protein interaction network was drawn for LGALS-11 and LGALS-14 affinity purified proteins specific to adult parasitic stage revealed that, LGALS-11 and LGALS-14 found to interact 5 unique proteins individually. Whereas nine proteins were found to interact with both LGALS-11 and LGALS-14 (Fig. 4). Carboxyl transferase, aldo keto reductase and myosin displayed unique interaction with LGALS-11. Whereas zinc metallopeptidase M13, porin domain containing protein, von Willebrand factor-like domain containing protein and mitochondrial solution substrate carrier protein displayed an interaction network unique to LGALS-14. Peptidase S28, alpha beta hydrolase fold-1, glutamate phenylalanine leucine valine dehydrogenase, nematode cuticle collagen, lipid transport protein containing vitellinogen and von Willebrand factor-like domains were found to interact both LGALS-11 and LGALS-14 (Fig. 4).

### Protease and phosphatase ligands

A significant number of proteins (*n* = 19) with enzyme activity were identified in adults, and similar proteins have been described in other ‘omic’ studies, suggesting that many of these enzymes of the protease family are conserved and evolutionarily related in nematodes (Campbell et al., 2011; Ghedin et al., 2007; Schwarz et al., 2013). A notable protease identified in the adult stage, is zinc metallopeptidase (M13 protease or neprilysin). Zinc metallopeptidases have been reported as the major protein fraction of host protective glycoprotein complex H-gal-GP (*Haemonchus* galactose containing glycoprotein). Another important metalloprotease is H11 family which has been shown to provide protection when used as vaccine antigen (Newton & Munn, 1999). Several studies isolated zinc metallopeptidases from crude extracts of *H. contortus* using lectins that have a binding preference to β-D-galactose and, following vaccination of sheep, led to reduced worm burdens following challenge infection (Dicker et al., 2014; Newlands et al., 2006; Smith et al., 1999; Smith et al., 2000). It appears inhibition of metalloproteases interferes with ability of *H. contortus* to digest its blood meal.

Another important group of proteases were found to interact with LGALS-11 and LGALS-14, which are S28 family (Table 1). Four different S28 proteases interacted were shown to interact with LGALS-11 and LGALS-14 in particular HCOI_01497800, HCOI_01562400 and HCOI_01624000 which have homology to contortin (Fig. S2). Contortin is intestinal antigen of *H. contortus* which induced significant levels of protection when used to vaccinate lambs (Munn, Greenwood & Coadwell, 1987). Contortin is comprised of two proteins, Hc-PCP1 and Hc-PCP2 that can degraded the C-terminal end of the fibrinogen alpha-chain therefore inhibiting blood clotting (Geldhof & Knox, 2008).

### Blood ligands

A number of parasite molecules were identified that interact with host galectins and are potentially involved in manipulating the host blood function in the adult stage but not larvae of *H. contortus*. That the adult stage of this nematode is primary a blood feeder may explain the lack of such molecules identified in the larvae. Blood feeding parasites are known to use several mechanisms to suppress platelet aggregation, allowing prolonged blood feeding by retarding blood clotting (Liu & Weller, 1992). The von-Willebrand factor (VWF) is a well-known protein reported in integrin and other extracellular proteins (Whittaker & Hynes, 2002). The binding of a C-type lectin (CLEC4M), with VWF has previously been shown to enhance the internalisation of VWF by the host cells and alter plasma levels of VWF (Rydz et al., 2013). In previous reports, proteins containing the VWF-like domain are localised in nematode intestine and suggested to play critical roles in cell adhesion and platelet aggregation (Wohner et al., 2012). A multimeric glycoprotein containing VWF-like domain was identified previously in adult *H. contortus* that can suppress platelet aggregation (Crab et al., 2002). In this study, a protein containing a VWF-like domain (W6ND82) was eluted from the LGAL14 column, which might suggest that this host galectin plays a role in potential modulating the ability of the parasite to suppress blood clotting. This protein was not detected in larval stage by both LGALS-11 and LGALS-14. However, the functional significance of VWF domain containing proteins in parasitised animals remains unknown, warranting further study.

### Specific sperm-coating protein (SCP)

The stage-specific sperm-coating protein (SCP) identified interacting with host galectins in this study are common to many nematode species (Cantacessi & Gasser, 2012) and are suggested to play critical roles in infection and immunomodulatory events such as neutrophil inhibition (Cantacessi et al., 2012; Gadahi et al., 2016; Hewitson, Grainger & Maizels, 2009). Transcriptomic studies of *H. contortus* have identified that 54 genes containing one or more SCP-like domains are upregulated in the blood-feeding adult, suggesting that SCP proteins have active and stage-specific involvement at the onset of blood feeding (Wang & Kim, 2003). Similar SCP domain containing proteins (Hc24 and Hc40) were reported in excretory/secretory proteins of *H. contortus* (Yatsuda et al., 2003). Although there is some information for SCP domain-containing proteins in *C. elegans* (O’Rourke et al., 2006; Wang & Kim, 2003), their biological functions in *H. contortus* needs experimental investigation.

## Conclusion

Recently, host galectins have been hypothesised to interact with molecules to modulate host-pathogen interactions in ruminants (Hoorens et al., 2011; Kemp et al., 2009; Preston et al., 2015b). The finding that LGAL-14 is concentrated within eosinophils (an immune cell considered a major mediator of parasite killing, including of *H. contortus*) suggested the possibility of a direct role for ruminant galectins in mediating parasite-killing (Meeusen & Balic, 2000; Robinson et al., 2011). The subsequent demonstration of direct binding of LGAL-11 to *H. contortus* and their ability to inhibit larval development and growth *in vitro* has confirmed the roles of galectins and ability to directly kill relatively large multicellular pathogens (Preston et al., 2015b).

The parasite surface is the key contact with the host and is often considered important source of potential vaccine molecules. Correspondingly, 45% of the glycoproteins that the two galectins bound were membrane proteins of the adult stage of *H. contortus,* and included vitelline, myosin and M13 protein (neprilysin); these proteins have been previously assessed as vaccine candidates (Knox, 2011; Strube et al., 2015; Tellam et al., 2002). This evidence would indicate that other putative glycoproteins identified here by these ruminant galectins might facilitate the identification of new intervention targets and, thus, warrant further investigation. In conclusion, the analysis of parasite proteins recognised by galectins that are involved in resistance to parasites (Guo et al., 2016; Preston et al., 2015a; Preston et al., 2015b), has identified several interesting stage-specific proteins. Exploring the possible biological roles and potential antihelminthic activities of these proteins has significant potential to advance our understanding of the host-parasite interplay and inform future parasite control strategies.

##  Supplemental Information

10.7717/peerj.4510/supp-1 Table S1 Identification by mass spectrometry of larval and adult *Haemonchus contortus* proteins eluted from control resin columns

10.7717/peerj.4510/supp-2 Figure S1 Schematic flow of pull-down experiment to identify the interactome Lysates of *Haemonchus contortus* (larval or adult worms) containing glycoproteins were isolated using immobilised recombinant LGALS-11 and LGALS-14 columns and eluted using a high concentration of *β*-D-galactose. The glycoproteins of larval and adult stages that interact with host galectins were analysed by LC-MS/MS. The spectra obtained from the LC-MS/MS were analysed using the Mascot (Perkins et al., 1999) and the NCBI protein database.

10.7717/peerj.4510/supp-3 Figure S2 Pair wise sequence alignment of S28 proteases with Hc-PCP1 and 2 *H. contortus* S28 protease proteins HCOI_01497800 (A), HCOI_01562400 (B) and HCOI_01624000 (C) were aligned with Hc-PCP1 or Hc-PCP2. The + sign represent conserved amino acid substitution.

## Additional Information and Declarations

### Data Availability

The following information was supplied regarding data availability: The mass spectrometry proteomics data have been deposited to the ProteomeXchange Consortium via the PRIDE partner repository with the data set identifier PXD008435 and DOI 10.6019/PXD008435.
