# Supplementary material for: Proteomic identification of galectin-11 and 14 ligands from Haemonchus contortus
Source: PeerJ. 2018 Mar 19;6:e4510. doi: 10.7717/peerj.4510 (PMC5863708; doi:10.7717/peerj.4510)
Supplement: Figure S1 — Lysates of Haemonchus contortus (larval or adult worms) containing glycoproteins were isolated using immobilised recombinant LGALS-11 and LGALS-14 columns and eluted using a high concentration of β-D-galactose. The glycoproteins of larval and adult stages that interact with host galectins were analysed by LC-MS/MS. The spectra obtained from the LC-MS/MS were analysed using the Mascot (Perkins et al., 1999) and the NCBI protein database. [file peerj-06-4510-s002.pdf]

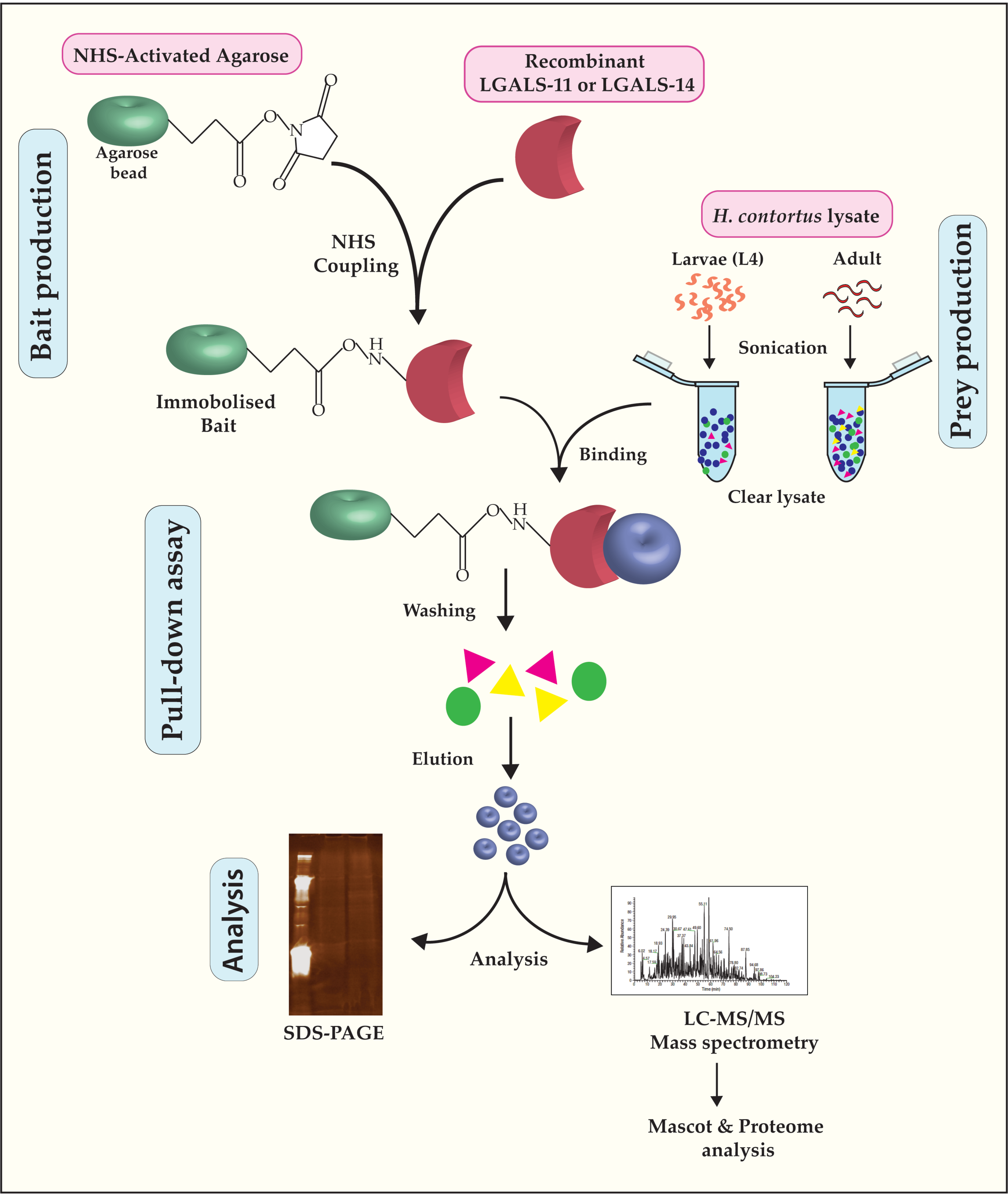

NHS-Activated Agarose
Recombinant LGALS-11 or LGALS-14
Agarose bead
Bait production
NHS Coupling
H. contortus lysate
Larvae (L4)
Adult
Sonication
Prey production
Immobolised Bait
Binding
Clear lysate
Pull-down assay
Washing
Elution
Analysis
SDS-PAGE
LC-MS/MS Mass spectrometry
Mascot & Proteome analysis
