# Supplementary figures and images for: Proteomic identification of galectin-11 and 14 ligands from Haemonchus contortus
Source: PeerJ. 2018 Mar 19;6:e4510. doi: 10.7717/peerj.4510 (PMC5863708; doi:10.7717/peerj.4510)

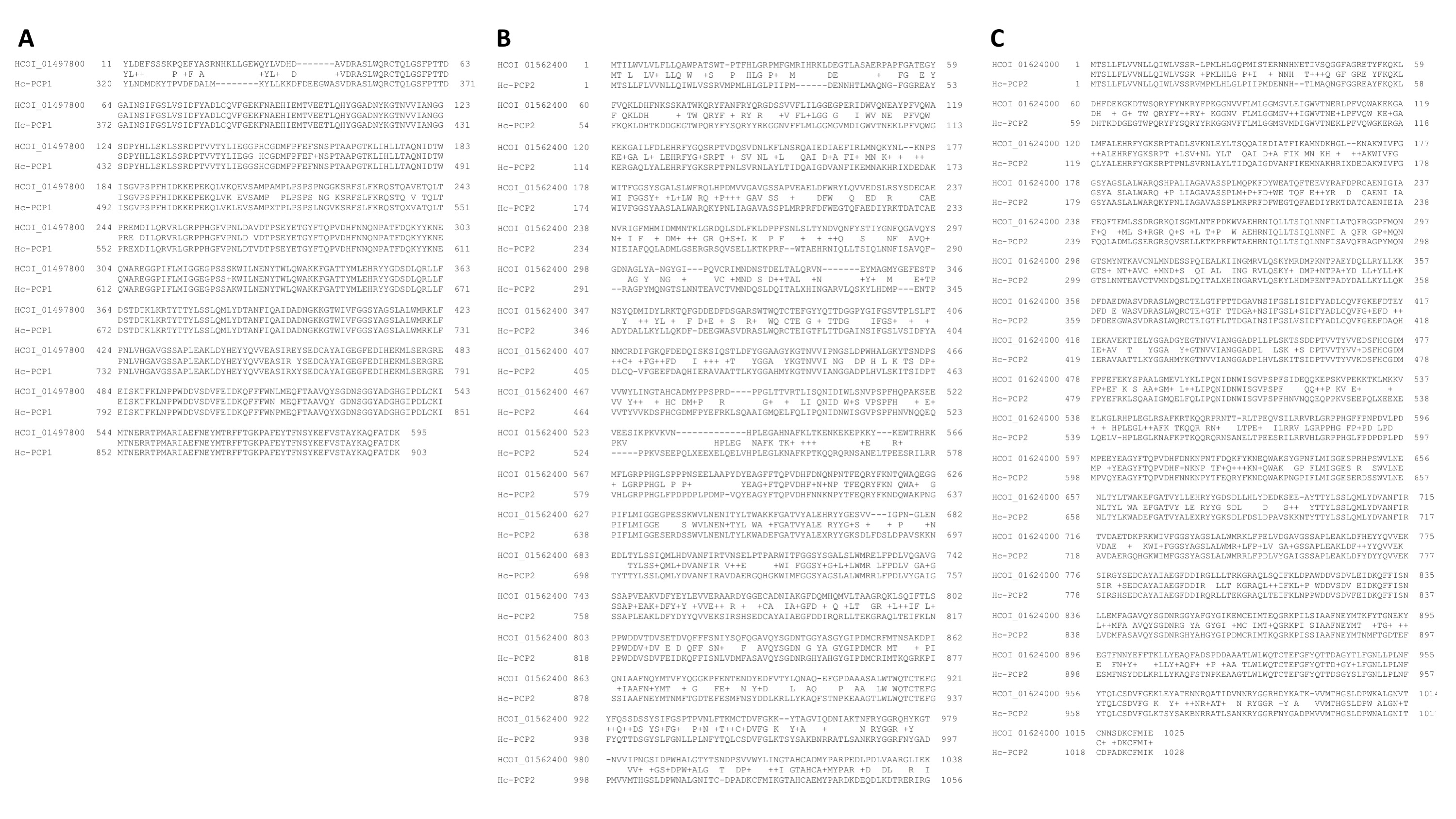

Supplement: Figure S2 — H. contortus S28 protease proteins HCOI_01497800 (A), HCOI_01562400 (B) and HCOI_01624000 (C) were aligned with Hc-PCP1 or Hc-PCP2. The + sign represent conserved amino acid substitution. [file peerj-06-4510-s003.jpg]
